# Dengue–SARS-CoV-2 interactions: immune crosstalk, variant emergence, and clinical outcomes

**DOI:** 10.3389/fimmu.2026.1650425

**Published:** 2026-02-05

**Authors:** Mariana Parra-González, Lucio Nájera-Maldonado, Esperanza Peralta-Cuevas, Ashley Gutierrez-Onofre, Luis A. Jaimes-López, Juan A. Juarez-Antonio, Nahomi Y. Degollado-Hernández, Igor Garcia-Atutxa, Francisca Villanueva-Flores

**Affiliations:** 1 Centro de Investigación en Ciencia Aplicada y Tecnología Avanzada (CICATA) Unidad Morelos, del Instituto Politécnico Nacional (IPN), Xochitepec, Mexico; 2 Universidad Tecnológica Emiliano Zapata del Estado de Morelos, Emiliano Zapata, Morelos, Mexico; 3 Universidad Católica de Murcia (UCAM), Departamento de Ciencias de la Computación, Murcia, Spain

**Keywords:** antibody-dependent enhancement, COVID-19, dengue, dengue–COVID-19 co-infection, immune crosstalk, syndemic dynamics

## Abstract

This review aims to provide an overview of dengue–COVID–19 co-infection, emphasizing recently described immunological, genomic, and eco-epidemiological interactions that may influence clinical outcomes and viral evolution. It brings together molecular evidence, immunological perspectives, and epidemiological insights to summarize current hypotheses and working models of these complex disease interactions. We summarize and critically discuss evidence on antibody-dependent enhancement (ADE), cross-reactive immune responses, and cytokine amplification pathways, and propose mechanisms that could underlie exacerbated disease severity. Published clinical data indicate heterogeneity in co-infection outcomes globally, from mild presentations to severe complications, such as hemorrhagic stroke, acute kidney injury, and increased mortality, particularly among populations with prior dengue exposure. Diagnostic complexities arising from serological cross-reactivity underscore the need for simultaneous molecular testing to ensure accurate pathogen identification. Additionally, we review current evidence on reciprocal selective pressures between SARS-CoV-2 variants and dengue serotypes, highlighting potential evolutionary impacts arising from their co-circulation. The available evidence suggests that co-infection may exacerbate inflammatory pathways, lead to increased vascular and organ damage, and complicate patient management. However, definitive clinical evidence for ADE remains inconclusive, underscoring an ongoing need for targeted mechanistic studies. By outlining significant knowledge gaps and summarizing proposed research directions, this review aims to provide a valuable reference for clinicians, immunologists, epidemiologists, and policymakers managing concurrent dengue and COVID-19 outbreaks.

## Graphical Abstract

**Figure:**
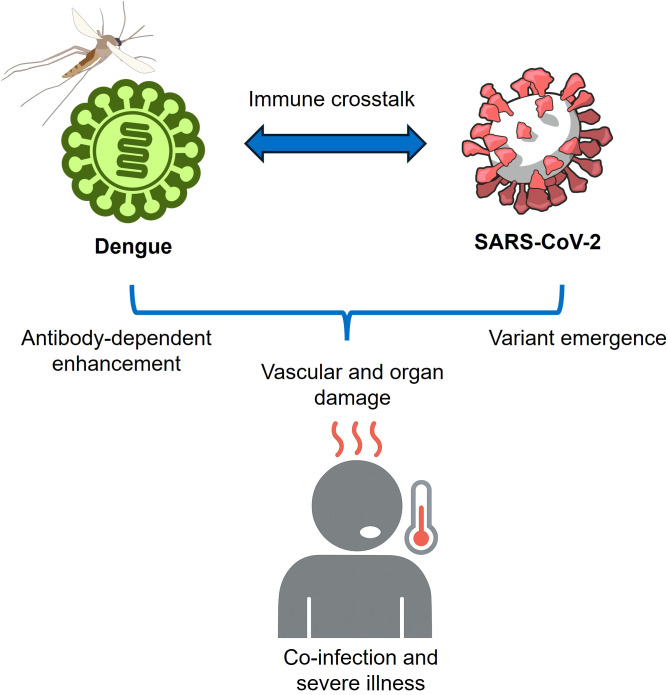

## Introduction

Dengue virus and SARS-CoV-2 (the cause of COVID-19) have imposed enormous global health burdens in recent years. Dengue, a mosquito-borne flavivirus, is endemic in over 100 countries and causes an estimated 300–400 million infections annually. Nearly half of the world’s population (about 4 billion people) lives at risk of dengue, predominantly in tropical and subtropical regions (1, 2). COVID-19, caused by a novel coronavirus that emerged in 2019, has spread worldwide with over 767 million confirmed cases and almost 7 million deaths to date (3, 4).

Emerging evidence suggests that dengue and COVID-19 are converging into a syndemic, characterized by overlapping epidemics that exacerbate each other’s impact, in regions where both viruses co-circulate. In tropical areas of Asia, Latin America, and Africa, COVID-19 surged alongside endemic dengue outbreaks, straining public health systems with a dual burden. A 2023 multi-continental analysis of weekly surveillance data from South America, Africa, and Southeast Asia reported that dengue and COVID-19 notifications rise and fall in lock-step, with statistically significant positive correlations (Pearson r ≈ 0.35–0.60) in Brazil, Peru, Colombia, Cambodia, and Kenya, even after controlling for humidity and temperature, suggesting shared climatic or behavioral drivers of transmission (5). Nations such as Brazil, Colombia, Peru, and Singapore have simultaneously faced surges of dengue alongside waves of COVID-19 (6–10).

The simultaneous transmission of these viruses presents challenges, including diagnostic confusion, strained healthcare resources, and potential interactive disease mechanisms (5, 11, 12). Understanding the molecular and immunological interplay between DENV and SARS-CoV-2 is crucial, as co-infections in respiratory diseases often increase clinical severity; for example, early reports showed a ~30% mortality in SARS-CoV-2–influenza co-infections, far exceeding mortality rates in COVID-19 alone (9). Dengue–COVID-19 co-infection is particularly concerning due to dengue’s risk of high fever, capillary leakage, and hemorrhagic complications, combined with COVID-19’s potential for severe pneumonia, coagulopathy, and multi-organ failure. Importantly, early in the pandemic, some reports that appeared to be “dengue–COVID-19 co-infections” were actually COVID-19 cases with false-positive dengue serology due to antibody cross-reactivity; those patients were not truly co-infected (13). To avoid any ambiguity, throughout this review, we use “co-infection” only for cases in which both viruses are laboratory-confirmed (e.g., DENV RT-PCR/NS1 and SARS-CoV-2 RT-PCR/antigen). This review examines molecular, immunological, and epidemiological interactions in dengue–SARS-CoV-2 co-infection, emphasizing mechanisms such as antibody-dependent enhancement (ADE), viral interference, and immune-driven viral evolution. Integrating immunological and genomic perspectives, we propose a mechanistic framework to inform public health strategies and guide future research on dual outbreaks.

## Clinical–immunological landscape of dengue–COVID-19 co-infection

Early in the pandemic, serological cross-reactivity led to misclassification of some SARS-CoV-2 infections, including those mistaken for dengue, complicating diagnosis and delaying care. Although systematic data remain limited, several studies now offer a clearer, though heterogeneous, picture. In Buenos Aires, a prospective case series identified 13 hospitalized adults positive for both SARS-CoV-2 (RT-PCR) and dengue (NS1/RT-PCR); none required intensive care or died (14). In contrast, a cross-sectional survey in Dhaka (2021–2023) found dual infection in 763 of 2,458 individuals tested (31%), peaking at 14% in densely populated areas (adjusted OR 2.4, 95% CI 1.9–3.0) (15). A Brazilian cohort during a dengue outbreak reported 25 acute dengue cases (6%) among 400 screened individuals (3). Complementing patient-level observations, Bergero et al. demonstrated fluctuations in COVID-19 incidence predicted dengue notifications up to four weeks in advance, suggesting an epidemiological connection (5).

The clinical severity of dengue–COVID-19 coinfection varies widely. In Dhaka, co-infected patients had a 4.1-fold higher risk of severe or very severe disease than those with COVID-19 alone (adjusted OR 4.12, 95% CI 3.05–5.54), often presenting acute cardiac injury (32%), acute kidney injury (49%), and cognitive impairment (22%) (15). A Brazilian series reported even greater severity: 95% hospitalization, 87% mechanical ventilation, and 50% mortality, increasing to 60% in patients with prior dengue exposure (3). These patterns suggest a possible synergistic pathogenic mechanism via cytokine amplification or antibody-dependent enhancement.

In contrast, Buenos Aires’ data showed no severe outcomes, with only mild or uncomplicated courses. An early hypothesis proposed that dengue-associated anticoagulant properties might offset COVID-19-induced thrombosis, though this remains speculative and untested (13, 14). Disparities among datasets, likely due to differences in study design, demographics, viral variants, and baseline immunity, underscore the need for standardized multicenter studies to clarify risk factors influencing outcomes.

**Table 1** summarizes confirmed dengue–COVID-19 co-infection cases (2020–2023), detailing patient demographics, diagnostic methods, clinical manifestations, immunological and laboratory markers, treatments, and outcomes. A total of 811 cases were reported from Bangladesh, Brazil, Colombia, the Philippines, Argentina, Thailand, Indonesia, India, and French territories (Reunion and Mayotte). Common presentations—high-grade fever, asthenia, myalgia, and thrombocytopenia overlapped for both diseases, complicating diagnosis and management. Severe complications were linked to hyperinflammatory states, possible immune-mediated pathology, and coagulopathy, occasionally causing fatal events such as strokes and multi-organ failure. Immunological profiles consistently showed lymphopenia, leukopenia, and thrombocytopenia, with elevated cytokines, D-dimer, and ferritin. Most patients recovered with supportive care, but serological cross-reactivity posed diagnostic challenges, emphasizing the need for simultaneous pathogen-specific molecular testing. These findings highlight the importance of dual-pathogen surveillance, early recognition, and targeted management to reduce complications and mortality in co-circulation regions.

**Table 1:** Global overview of confirmed dengue–COVID–19 co-infections: clinical, immunological, and epidemiological insights.

Region	Study period	Patient’s age and sex	No. of cases (Confirmed)	Diagnostic methods	Clinical features	Key lab findings	Treatment strategy	Clinical outcome	Remarks	Study type	Reference
Reunion (France)	April 2020 (pandemic wave)	18-year-old male	1 (confirmed)	Dengue: RT-PCR (DENV-1) and IgM+; COVID-19: RT-PCR	High fever, asthenia; later rash with mild hemorrhagic signs (gingival bleed); no pneumonia on CT.	Thrombocytopenia (~1.06×10^5^/μL), leukopenia (1.7×10^3^/μL), lymphopenia (0.6×10^3^/μL); mildly elevated AST. CRP normal.	Supportive care (antipyretics, hydration); discharged initially, readmitted for bleeding.	Recovered	First Réunion Island co-infection; highlighted overlapping features, emphasizing confirmatory testing to avoid misdiagnosis.	Case Report	(16, 17)
Mayotte (France)	(March 2020). (early pandemic)	44-year-old male (traveler)	1–2 (confirmed)	Dengue: NS1 antigen and IgM positive; COVID-19: RT-PCR (assumed from context)	Acute febrile illness; initial dengue-like symptoms, later respiratory (COVID-19).	(Not reported in detail, NI) Likely thrombocytopenia and leukopenia (common in dengue), NI in source.	Supportive dengue care; COVID-19 isolation (oxygen if needed).	Recovered (index case)	First confirmed Mayotte co-infections; emphasized dual infection possibility and outbreak awareness in island settings.	Case Report	(18)
Brazil (São Paulo)	April 2020	25-year-old male	1 (confirmed)	Dengue: RT-PCR positive; COVID-19: RT-PCR.	2-day asthenia, headache, arthralgia, high fever; no respiratory distress.	Mild thrombocytopenia, leukopenia, elevated liver enzymes (mild AST/ALT); consistent with dengue/COVID (NI exact values).	Supportive (fluids, antipyretics); monitored for complications.	Recovered (favorable outcome)	First reported Argentina co-infection; overlapping symptoms and laboratory results, with a favorable outcome; emphasized the importance of dual testing.	Case Report	(19, 20)
Brazil (Brasília)	March–April 2020	56-year-old female	1 (confirmed)	Dengue: RT-qPCR, NS1 and IgM/IgG all positive; COVID-19: RT-PCR positive (nasopharyngeal swab).	12-day illness: fever, sore throat, anosmia/ageusia, headache, mild dyspnea; dengue rash, gastrointestinal symptoms in hospital (no severe signs).	Leukopenia (WBC 3.7→1.5×10³/μL), initial lymphopenia; mild thrombocytopenia (~1.6×10^5^/μL, day 3); elevated D-dimer (~3986 ng/mL), ferritin (~559 μg/L); mild AST/ALT rise. Bilateral ground-glass opacities (COVID pneumonia).	Empiric COVID therapy, anticoagulation, and supportive care for dengue (fluids, monitoring).	Recovered (discharged after 6 days)	First SARS-CoV-2/DENV co-infection in Brazil; emphasized COVID-19 diagnosis does not exclude dengue (and vice versa); overlapping labs complicated diagnosis.	Case Report	(19)
Brazil (São Paulo)	July 2020	38-year-old male doctor (plus family)	4 (cluster, confirmed)	Dengue: NS1 antigen positive (all family); COVID-19: RT-PCR confirmed (doctor and family).	The doctor and family initially had dengue (fever, myalgias), then COVID-19 (the doctor was likely an asymptomatic carrier). No severe complications reported.	(NI, individual lab data not detailed; presumed dengue labs: low platelets, etc., in family; doctor’s COVID likely confirmed by PCR.)	Supportive care for dengue; isolation once COVID-19 is diagnosed.	All recovered (none severe)	Illustrates co-epidemic: dengue outbreak overlapping COVID-19 surge; healthcare workers contracted dengue, then SARS-CoV-2 abroad, infecting family. Highlights HCW transmission risk, spatial surveillance need.	Case Report + Spatial Analysis	(21)
**Brazil (São Paulo)**	June 2020	45-year-old male (ICU patient)	1 (confirmed)	Dengue: RT-PCR positive; COVID-19: RT-PCR positiveresearchgate.net	Presented initially with acute stroke (CVA), fever, and viral symptoms; later developed ARDS.	(Limited details) Marked inflammatory markers due to COVID-19; dengue confirmed by PCR post-mortem. Stroke is attributed to co-infection hypercoagulability.	Intensive care (including ventilation and critical care); supportive measures.	Fatal (hemorrhagic stroke)	First reported COVID–19–dengue co-infection with stroke; highlights severe complications (stroke, ARDS) from immune/inflammatory interactions (coagulopathy).	Case Report	(22)
Brazil (Bahia)	may-20	39-year-old male	1 (confirmed)	Dengue: RT-PCR positive (DENV); COVID-19: RT-PCR positive.	Fever (2 days), myalgia, diarrhea, and ageusia. Initially diagnosed as dengue, five days later, he also tested positive for COVID-19 (contact with infected mother). Mild dyspnea developed, no severe complications.	(No baseline labs reported in case letter; presumably thrombocytopenia was present since treated as dengue; no lab work due to resource limits)	Symptomatic treatment for dengue (analgesics, antipyretics); strict home isolation for COVID-19. Analgesics were adjusted when myalgia worsened.	Recovered (outpatient follow-up)	Demonstrated dual epidemic risk: field investigation found Aedes mosquitoes positive for DENV in the patient’s community. The case highlights the importance of vigilant surveillance and confirms that co-infection can occur even during the early stages of an outbreak.	Case Correspondence	(23)
Thailand (Bangkok)	(Mar 2020). (early outbreak)	50-year-old female	1 (confirmed)	Dengue: Rapid test (NS1-Ag faint + IgG); DENV-2 confirmed by RT-PCR. COVID-19: RT-PCR positive (NP/throat swab).	Acute fever (39 °C) with myalgia, nausea, vomiting for 1 day. No cough or dyspnea. *Laboratory-confirmed* dengue without warning signs concurrent with asymptomatic-to-mild COVID-19.	Leukopenia (WBC 3.7→1.5×10^3^/µL/µL) with lymphopenia (222/µL) on admission; normal platelets initially, pmc.ncbi.nlm.nih.gov, slight drop later (~1.58×10^5^/µL/µL). Elevated AST/ALT (up to ~136 U/L ALT). No chest X-ray infiltrates are present throughout.	Supportive only: hydration, antipyretics, and close monitoring. Isolated in the COVID ward while managing dengue fever.	Recovered (fever resolved <48h; discharged by day 12)	Coinfection was identified during a routine dengue workup in a returning traveler (a flight attendant). Showed how overlapping lab features (leukopenia, transaminitis) complicated the diagnosis. Emphasized the need to test for both viruses in AUFI in the tropics.	Case Report	(24)
Thailand (Nonthaburi)	mar-20	35-year-old male + cluster	1 (confirmed index + 2 secondary COVID)	Dengue: IgM/IgG positive (acute secondary dengue); COVID-19: RT-PCR positive.	Initially diagnosed as dengue fever (high fever, mild thrombocytopenia, positive Dengue IgM/IgG). Later confirmed SARS-CoV-2 infection; patient remained mild. Transmitted COVID-19 to a nurse (35F) and his 3-year-old daughter.	Mild thrombocytopenia (NI exact count); no severe lab derangements reported. *False-positive dengue serology* was initially considered, but true co-infection was confirmed.	Supportive for dengue, COVID-19 isolation, and contact tracing.	Recovered (index case); the nurse and child also recovered.	Demonstrated a misdiagnosis pitfall: A COVID-19 case was misidentified as dengue initially. Highlighted risk of nosocomial spread, a nurse infected while caring for a dengue-suspected patient who had unrecognized COVID-19. Raised awareness for dual testing in endemic areas.	Case Study	(25)
Philippines (Laguna)	2021(during co-epidemic)	38-year-old male	1 (confirmed)	Dengue: Duo rapid test IgM(+) & IgG(+); COVID-19: RT-PCR positive. (No PCR for dengue reported.)	High-grade fever with nausea, arthralgia, and myalgia. No cough or respiratory symptoms. Moderately ill but no severe complications; prompt recovery.	Mild lab changes: persistent high fever but no severe thrombocytopenia noted (NI exact counts), classified as “moderate to severe” by test criteria.	Supportive treatment only; symptoms improved rapidly.	Recovered (rapid defervescence and recovery)	Illustrates difficulty in clinical distinction: in dengue-endemic Philippines, COVID-19 cases had positive dengue serology. Emphasizes the need for concurrent diagnostic testing to prevent missed co-infections.	Case Report	(26)
Bangladesh (Dhaka)	Dec 2021 – Nov 2023	10–69 years (median ≈30s); ~58% M	763 (confirmed co-infections)	Dengue: NS1 antigen and IgM serology; COVID-19: RT-PCR and antigen tests.	Common symptoms: fever (100%), weakness (~90%), chills (~82%), fatigue (~81%), headache (~81%), and overlapping symptoms between infections. Co-infection had higher severe rates: cardiac damage (31.6%), kidney injury (49%).	Labs: Frequent thrombocytopenia, lymphopenia in co-infections. Elevated inflammatory markers; 4-fold higher severe disease risk vs single infections. Cytokines NI.	Integrated protocols: dengue-specific fluid management, COVID-19 antivirals as indicated, supportive care, complication monitoring.	Worse outcomes than mono-infection: co-infected patients had significantly higher odds of severe illness (OR ~4.2). The overall mortality rate (NI) was low in the study.	Largest COVID–dengue co-infection study; risk factors: age >50, high density, prior dengue. Suggested possible immune enhancement (unproven ADE), urged diagnostic vigilance during concurrent outbreaks.	Cohort Study	(15)
Argentina (Buenos Aires)	Mar–Jun 2020	Adults (18–69 years); 8 M/5 F	13 (confirmed)	Dengue: PCR and/or NS1 antigen confirmed; COVID-19: RT-PCR confirmed.	All had acute fever on admission. Symptoms: headache (most cases), 8/13 with cough or other respiratory symptoms, 5 with pneumonia on CXR, 3 with dengue-like rash. None had shock or MIS.	Nearly all had lymphopenia at presentation; some had mildly elevated liver enzymes (NI); typical dengue labs (hemoconcentration, thrombocytopenia) were mild.	Supportive care in the hospital for both illnesses (antipyretics, IV fluids, oxygen for pneumonia as needed). No ICU care needed.	All survived; no ICU admissions, zero deaths.	First cohort study of dengue–COVID co-infections. Outcomes were no worse than those with a single infection, suggesting no additive mortality in otherwise healthy patients. Reinforced that co-infection can occur during simultaneous outbreaks and stressed the need for dual testing in endemic regions.	Retrospective Case Series	(14)
**Northern Peru**	Oct-Nov 2020	45-year-old male	1 (COVID-19 and Dengue serotype 1).	RT-PCR (nasopharyngeal swab for SARS-CoV-2, blood for dengue virus).	Fever, general discomfort, arthralgia, retro-orbital pain, abdominal pain, anosmia, ageusia, dry cough, dyspnea, pruritus.	Leukocytosis, then normalization, elevated inflammatory markers (PCR, ferritin, D-dimer, LDH), and initial creatinine elevation.	Intravenous fluids (saline solution 0.9%), oxygen therapy (nasal cannula at 3 L/min), and a single dose of Tocilizumab (400 mg IV). Avoidance of corticosteroids and anticoagulants.	Favorable recovery, discharged after 12 days without adverse events.	Rare simultaneous critical phase of dengue and hyperinflammatory phase of COVID-19.	Case Report	(27)

To clearly illustrate these clinical-immunological relationships, **Table 2** summarizes clinical manifestations observed in dengue–COVID–19 co-infections, proposed molecular mechanisms, supporting experimental evidence, and existing gaps. The analysis reveals several insights: (1) severe clinical outcomes likely arise from synergistic interactions between dengue virus and SARS-CoV-2 at molecular and immunological levels; (2) antibody cross-reactivity significantly complicates diagnosis, reinforcing simultaneous molecular testing; and (3) although antibody-dependent enhancement (ADE) is plausible in co-infection, definitive clinical evidence is lacking, highlighting the need for targeted mechanistic investigations. Together, these findings underscore dengue–COVID-19 co-infection as an amplified clinical threat characterized by intricate immunopathological interactions, necessitating integrated clinical approaches and multidisciplinary research.

**Table 2:** Clinical observations in dengue–COVID–19 co-infection and proposed mechanisms.

Clinical observation	Proposed molecular/immunological mechanism	Experimental evidence	Limitations/gaps	References
Diagnostic confusion due to overlapping features *(e.g., fever, myalgia, thrombocytopenia; false-positive dengue tests in COVID-19 patients)*	Both diseases share symptoms (fever, cytopenias, capillary leak), and antibody cross-reactivity can cause false-positive dengue tests. Overlapping features, such as vascular leak and coagulopathy, further complicate the clinical diagnosis.	Cases show COVID-19 patients initially misdiagnosed due to false-positive dengue serology. Both infections cause capillary leakage, thrombocytopenia, and coagulopathy, complicating diagnosis.	Primarily based on limited reports, the extent of serological cross-reactivity remains unclear. Clinical vigilance and PCR *Modeling show that single-pathogen testing for both viruses is* essential, though controlled trials on diagnostic strategies are lacking.	(28–30)
Severe disease outcomes (shock, ARDS, multi-organ failure, high mortality)	Co-infection may amplify hyperinflammation and tissue damage, triggering severe cytokine storms and endothelial injury (lungs, vasculature). This leads to ARDS, shock, and organ failure. Both viruses might excessively activate complement and coagulation pathways.	A systematic review linked dengue–COVID co-infection to severe disease and increased mortality. A Brazilian cohort reported >85% invasive ventilation use and ~50–60% mortality, exceeding COVID-19 alone. Patients often had respiratory failure alongside dengue hemorrhagic symptoms.	Evidence is observational and may favor more severe cases. Mild cases in some regions (e.g., Argentina) suggest outcomes vary by virus or host factors. Direct evidence is absent; hypotheses, such as dengue’s anticoagulant effect on COVID-19, remain untested.	(3, 16)
Exacerbated inflammatory (“cytokine storm”) response	Both viruses activate innate immune pathways (e.g., Toll-like receptors, NLRP3 inflammasome) and stimulate immune cells, triggering the release of extensive cytokines. Co-infection amplifies cytokine levels (IL-1β, IL-6, TNF-α, and IL-10), increasing vascular permeability and tissue damage beyond that of single infections.	In co-infected patients, levels of IL-1β, IL-10, and TNF-α were significantly higher, indicating intense inflammation. Both dengue and SARS-CoV-2 individually trigger cytokine storms, inflammasome activation, and elevated IL-6, leading to endothelial permeability, acute respiratory distress syndrome (ARDS), or shock. This suggests additive cytokine effects during co-infection.	Direct comparisons between co- and single-infection cytokine levels are scarce. Evidence of amplified cytokines in co-infection remains primarily correlative, and it’s uncertain whether the effects are synergistic or additive. Variability among patients is high, studies are small, and no intervention studies have confirmed the benefits of reducing combined cytokine responses.	(3, 30)

The immunopathogenesis of dengue–COVID-19 co-infection remains unclear. Dengue typically induces a TNF-α-driven cytokine storm, whereas severe COVID-19 involves hyperinflammation and coagulopathy (3, 31). In co-infections, these pathways may converge or amplify. In Brazil, co-infected patients showed significantly higher levels of IL-1β, TNF-α, and IL-10 than those with SARS-CoV-2 alone, suggesting a combined inflammatory response that may worsen vascular damage and organ dysfunction (3, 31). Reciprocal immune interactions have also been reported: antibodies to the SARS-CoV-2 spike protein receptor-binding domain can bind dengue proteins, inhibiting dengue replication and reducing vascular damage in murine models (32). Serum from COVID-19 convalescent patients neutralized dengue virus *in vitro*, implying potential cross-protection. However, dengue antibodies also cross-react with SARS-CoV-2 antigens, complicating serological diagnosis and raising the theoretical risk of ADE in co-infections (13). Although no clinical evidence supports the notion that ADE worsens COVID-19, the risk warrants investigation. Public health challenges include diagnostic complexity and disrupted control measures. In dengue-endemic areas, overlapping symptoms, fever, myalgia, and headache necessitate simultaneous molecular or antigen-based testing for both pathogens, as clinical and routine laboratory parameters lack specificity. Modeling shows single-pathogen algorithms miss many co-infections. Pandemic-related disruptions to vector control and increased household water storage boosted mosquito breeding, causing dengue spikes in several countries despite global declines (33). Machine-learning analyses linked dengue resurgence to mobility rebounds during peak transmission periods, as seen with concurrent dengue and COVID-19 surges in Latin America (5).

Given the regional variability in impact, robust multicenter studies integrating detailed clinical, immunological, and epidemiological data are urgently needed to guide public health strategies for dengue–COVID-19 co-infection. Addressing data limitations is essential for advanced analyses such as machine learning. The clinical data in **Table 1**, mainly isolated case reports and one larger study, lack the volume and diversity required for reliable predictive modeling. Future machine learning or computational studies will require significantly larger, more heterogeneous datasets and clearly defined analytical objectives.

## Antibody-dependent enhancement in dengue and coronavirus

ADE is a mechanism in which antibodies at sub-neutralizing levels bind to virus particles, paradoxically enhancing their entry into Fcγ receptor–bearing cells and leading to increased infection rather than protection. In dengue virus (DENV) infections, ADE is implicated in the development of severe disease. Classic experiments by Halstead and O’Rourke (34). demonstrated that non-neutralizing dengue antibodies render normally non-permissive leukocytes susceptible to infection, yielding more than 1,000 plaque-forming units per 10^6 cells (vs. near-zero without antibody). Strikingly, in one case, an enhancing effect was observed even at a 1:320,000 serum dilution (34).

At the clinical level, secondary heterologous DENV infections (with pre-existing antibodies from a prior infection) are far more likely to be severe; a meta-analysis found the relative risk of dengue hemorrhagic fever/shock syndrome in secondary infections compared to primary infections is ~23.7 (95% CI, 15–37) (35). Consistent with the ADE model, patients experiencing secondary DENV-2 infection have significantly higher viremia (mean ~11.6 log_10_ genome copies/mL) than those with primary infections (~10.6 log_10_) (36). Moreover, enhanced infection correlates with hyperactivated immune responses: severe dengue cases exhibit a “cytokine storm” profile (elevated IL-6, IL-8, TNF-α, etc.) alongside anti-inflammatory IL-10, which in one cohort averaged ~292 pg/mL in severe dengue versus ~157 pg/mL in milder cases (37, 38). These quantitative findings firmly establish ADE as a driver of dengue immunopathogenesis.

Whether ADE occurs with SARS-CoV-2 has been closely studied, given precedents in other coronaviruses (e.g., SARS-CoV-1, MERS-CoV). *In vitro* evidence shows SARS-CoV-2 can evade antibodies under certain conditions. Wang et al. (39) found that two neutralizing monoclonal antibodies (MW01, MW05) enhanced viral entry into FcγRIIB-expressing B cells using a SARS-CoV-2 pseudovirus. This occurred via bivalently bound spike–antibody immune complexes binding the inhibitory FcγRIIB receptor, enabling “Trojan horse” virus uptake—an effect abolished by Fc-region mutations that prevent FcγR binding (40). These findings suggest that SARS-CoV-2 could, in theory, exploit ADE-like pathways similar to those of flaviviruses. However, no *in vivo* evidence shows ADE at a meaningful scale in COVID-19. Neither natural infection data nor vaccine trials indicate worsening of disease due to pre-existing antibodies; COVID-19 lung pathology is instead driven by direct cytopathic effects and inflammation, not aberrant Fc-mediated uptake (41). While immunologists remain alert to ADE risks, especially with emerging variants and shifting antibody landscapes, the clinical consensus is that antibody-mediated enhancement has not significantly influenced COVID-19 severity in humans.

The ADE paradigm remains relevant in the context of dengue and COVID-19. In dengue-endemic regions such as Latin America and Southeast Asia, high dengue seroprevalence raises questions about cross-reactivity with SARS-CoV-2. Immunological cross-talk between DENV and SARS-CoV-2 has been documented. In India, 93% of SARS-CoV-2 seropositive individuals had dengue-reactive antibodies by rapid test or ELISA; notably, many COVID-19 convalescent sera neutralized dengue virus *in vitro*, despite 57% of donors lacking prior dengue infection (no DENV NS1 antibodies) (42). This suggests SARS-CoV-2 can elicit cross-reactive antibodies capable of inhibiting DENV.

Conversely, the impact of pre-existing dengue antibodies on COVID-19 has been examined epidemiologically. While true ADE of SARS-CoV-2 by dengue antibodies remains unproven, clinical observations are notable. In a Brazilian cohort during concurrent outbreaks, ~6% of hospitalized COVID-19 patients had acute dengue co-infection, with ~94.9% hospitalized, ~50% mortality, and an even higher fatality (~60%) plus more respiratory complications in those with past dengue immunity (secondary dengue). Such poor outcomes may reflect additive pathology but warrant vigilance (3).

In tropical regions, misdiagnosis occurs due to cross-reactive serology, and clinicians must distinguish overlapping dengue–COVID-19 symptoms (3). Overall, ADE illustrates how pre-existing immunity can paradoxically affect another pathogen. The DENV–SARS-CoV-2 interplay, ranging from possible cross-protection to theoretical enhancement, underscores the need for close monitoring during co-epidemics and continued research to assess any public health risk as both viruses co-circulate.

## Selective pressures and viral evolution in dengue–COVID-19 co-infections

### General mechanisms and immune pressures

When both DENV and SARS-CoV-2 infect the same host, their respective immune responses can intersect, potentially amplifying pathological outcomes. In a recent Brazilian cohort, patients co-infected with dengue and COVID-19 exhibited significantly elevated circulating levels of IL-1β, TNF-α, and IL-10 compared to those infected solely with SARS-CoV-2 (3). These findings indicate the concurrent activation of pro-inflammatory and anti-inflammatory pathways, which could exacerbate vascular injury and increase the risk of multi-organ dysfunction.

Immune crosstalk occurs bidirectionally. *In vitro* and murine studies show antibodies to the SARS-CoV-2 spike receptor-binding domain cross-react with DENV E and NS1 proteins, suppressing dengue replication and reducing hemorrhage and vascular permeability (32). Human COVID-19 convalescent sera also neutralize DENV in cell culture, suggesting prior SARS-CoV-2 infection or vaccination may confer partial dengue immunity. Conversely, DENV-induced antibodies cross-react with SARS-CoV-2 antigens, complicating diagnostics and raising theoretical ADE concerns. While no clinical evidence links pre-existing dengue immunity to worse COVID-19 outcomes (13), the risk remains unresolved. This interplay of cross-neutralization, possible ADE, and convergent inflammatory signaling requires further study across diverse epidemiological and genetic contexts.

### Public health and diagnostic implications

From a public health perspective, dengue–COVID-19 co-infection burdens both diagnosis and control efforts. In endemic areas with active SARS-CoV-2 transmission, clinicians should suspect co-infection in febrile patients due to overlapping symptoms (fever, myalgia, headache, rash) (13) and shared lab findings (leukopenia, thrombocytopenia, elevated transaminases). Accurate diagnosis demands simultaneous molecular or antigen-based testing (RT-qPCR or rapid antigen for SARS-CoV-2; NS1 antigen or RT-qPCR for dengue within five days), followed by IgM/IgG serology. Modeling and empirical analyses suggest that diagnostic strategies focused on a single pathogen can miss a substantial fraction of co-infections (43–45).

### Epidemiological and ecological context

The COVID-19 pandemic severely disrupted global efforts to control vector-borne diseases. Border closures, lockdowns, and movement restrictions halted entomological surveillance and community vector control, while prolonged household water storage during stay-at-home orders created ideal breeding sites for Aedes. Although many countries reported >40% declines in dengue notifications in 2020, sharp increases occurred in Peru, Singapore, and parts of India (33). Machine-learning and statistical analyses of dengue notifications, human mobility data and COVID-19 non-pharmaceutical interventions from multiple endemic countries suggest that dengue resurgence often follows rebounds in human mobility during warm, humid periods (33, 46–48).

Given that dengue and COVID-19 disproportionately affect low-resource settings, simultaneous outbreaks place severe strain on healthcare infrastructure, including hospital beds, intensive care units, and laboratory testing capacity. Immediate public health priorities should therefore include reinstating comprehensive vector control programs, integrating arbovirus and respiratory disease surveillance systems, and deploying cost-effective multiplex rapid diagnostic tests. Such integrated measures are essential to mitigate the combined clinical and epidemiological impacts of dengue–COVID–19 co-infection (6, 49, 50).

Current evidence unequivocally demonstrates that dengue–COVID–19 co-infection occurs and can significantly exacerbate disease outcomes; however, the clinical impact varies widely depending on local contexts. Some cohorts report notably high mortality rates approaching 50% (3), while others document mild or uncomplicated recoveries (14). Cross-reactive antibodies further complicate the diagnostic landscape but may also confer partial immune protection against subsequent infections (51). These divergent findings underscore critical knowledge gaps in pathogenesis, host immune response variability, and healthcare system resilience. Additionally, emerging epidemiological models highlight population-level factors, including holiday travel patterns, climatic anomalies, and rebounds in human mobility, synchronizing dengue and SARS-CoV-2 transmission cycles. Thus, future preventive strategies should integrate robust vector control measures, comprehensive surveillance of respiratory viruses, and targeted behavioral interventions (5).

As dengue continues its geographic expansion and SARS-CoV-2 transitions to endemicity, instances of dual infections are likely to increase. Therefore, elucidating the clinical trajectories, immunological interactions, and optimal management strategies for dengue–COVID–19 co-infection is an urgent priority (52). This is essential not only for effective clinical triage but also for informing policy decisions in resource-limited regions disproportionately affected by these overlapping health crises. The review presented here synthesizes current insights on the epidemiology, clinical severity, immunopathogenesis, and diagnostic challenges associated with co-infection. It outlines critical research directions to address this emerging global health challenge.

### DENV evolutionary dynamics under host immunity

Co-infection and pre-existing immunity exert intense selective pressures, driving pathogens to adapt to their host’s immune landscape. When two viruses circulate simultaneously, each can evolve in response to immune pressures shaped by the other. Dengue virus (DENV), with four antigenically distinct serotypes, illustrates this dynamic: infection by one serotype induces durable homotypic immunity via serotype-specific neutralizing antibodies, forcing each serotype to evade these antibodies or risk displacement. A 20-year analysis of 1,944 DENV isolates from Thailand (1994–2014) revealed consistent antigenic drift across all serotypes, reflecting adaptation to herd immunity, and transient antigenic convergence coinciding with epidemic peaks (53). Such convergence may enable viral lineages to exploit partial cross-immunity, boosting their competitiveness and driving larger outbreaks.

Notably, Thailand’s largest dengue epidemics were characterized by low antigenic diversity, with a single lineage that was adept at evading prevailing population immunity. Conversely, periods of lower transmission intensity exhibited greater antigenic diversity, indicating that no single variant possessed a significant immunological advantage. This cyclical pattern highlights the intricate interplay between viral evolution and host immune pressure: DENV serotypes regularly diversify antigenically to circumvent sterilizing immunity yet intermittently converge to exploit the residual cross-reactivity generated by preceding serotypes. This ongoing dynamic can be viewed as an immunological “arms race,” wherein viral adaptation and host immune responses continually influence each other’s evolution (53).

The phenomenon of antibody-dependent enhancement (ADE) in dengue further imposes distinct selective pressures on viral evolution. During secondary dengue infections, pre-existing heterotypic antibodies bind to a different DENV serotype without fully neutralizing it, facilitating enhanced viral entry into Fcγ-receptor-bearing cells. This process results in increased viral replication, elevated viral loads, and heightened immunopathology (53). In regions such as Bangkok, approximately 90–95% of hospitalized dengue patients experience secondary or subsequent dengue virus (DENV) infections, highlighting the prevalence of immune-primed populations that frequently encounter the virus. Consequently, DENV has evolved not only to evade existing immune responses but also to exploit partial cross-reactivity, thereby enhancing replication and transmission (54, 55).

During high dengue transmission, variants with optimal antigenic divergence (distinct enough to evade neutralizing antibodies yet similar enough to exploit ADE) gain a selective advantage and may quickly dominate. This balance reflects a trade-off between immune evasion and use of cross-reactive antibodies, creating a genetic and antigenic “arms race.” Over time, DENV serotypes accumulate antigenic changes to escape homotypic immunity, but antigenic mapping shows transient convergence, where lineages regain partial similarity to exploit cross-reactive antibodies and boost transmission (53). These quantitative findings illustrate clearly how host immune pressures drive the continual evolutionary turnover and antigenic modulation observed in dengue virus populations.

### SARS-CoV-2 variant evolution and DENV cross-reactivity

SARS-CoV-2 has likewise evolved under strong host immune pressures, with variants of concern (Alpha, Beta, Delta, Omicron, and sublineages) adapting mainly to evade neutralizing antibodies, and to a lesser extent, T-cell responses from prior infection or vaccination (56, 57). In immune populations, antibody escape is a key driver of variant success. In dengue-endemic regions, SARS-CoV-2 co-circulation with DENV may introduce additional selective pressures through cross-reactive immunity.

Mallick and Biswas (10) report that successive SARS-CoV-2 variants have acquired spike protein mutations within regions targeted by dengue-induced cross-reactive antibodies, reducing binding affinity. From Alpha (B.1.1.7) and Delta (B.1.617.2) to Omicron sublineages (BA.1, BA.2), amino acid substitutions consistently overlapped with dengue antibody epitopes. Omicron variants (2022–2023) showed ≥50% lower cross-reactivity to dengue-positive sera than earlier variants, confirmed by *in vitro* assays demonstrating reduced binding of dengue-immune human sera to mutated spike epitopes. These data suggest SARS-CoV-2 has accumulated antigenic changes to evade dengue-elicited antibodies, reflecting evolutionary pressure in dengue-experienced populations (10). Reciprocal selective pressures may allow dengue and SARS-CoV-2 to influence each other’s antigenic evolution, potentially producing variants with greater immune escape, transmissibility, or altered virulence. This underscores the need for integrated surveillance addressing the interactive evolutionary dynamics of co-circulating viruses (10, 56).

In overlapping dengue and COVID-19 epidemics, selective pressures may act on both viruses at the population and within-host levels. The immune environment shaped by one pathogen can influence the other’s evolution. In hosts with dengue immunity, SARS-CoV-2 may encounter cross-reactive antibodies or memory T cells that favor variants able to evade them; similarly, DENV infecting COVID-convalescent hosts may face coronavirus-induced immunity. While direct evidence of mutations from dengue–COVID interactions is lacking, analogous co-infections suggest plausibility. Immune cross-reactivity could drive “escape” from shared epitopes. SARS-CoV-2 variants with mutations in DENV-like regions, or DENV variants evading anti-Spike antibodies, may gain a fitness advantage. This remains speculative but is a key evolutionary question.

Multiple studies have explored how different SARS-CoV-2 variants cross-react with the four dengue virus serotypes. For example, Nath et al. (42) found that COVID-19 convalescent sera from early in the pandemic (Wuhan strain period) frequently showed dengue cross-reactivity: 93% of SARS-CoV-2 antibody–positive samples gave positive results in dengue serology, and these sera could neutralize DENV-1 *in vitro* (42). Recent investigations by Mallick & Biswas (10) compared antibodies from patients infected by various SARS-CoV-2 variants against all four DENV serotypes. Notably, they report that antibodies generated during earlier waves (e.g. the original Wuhan strain and Delta variant) were broadly cross-reactive and could neutralize all four DENV serotypes (58, 59). By contrast, antibodies from Omicron-wave infections showed much narrower dengue cross-reactivity (58, 59). For instance, Omicron-era COVID-19 sera in one study retained some cross-neutralizing activity against DENV-1, DENV-2, and DENV-4, but failed to neutralize DENV-3 effectively. This aligns with the observation that post-Omicron COVID-19 antibodies are less cross-reactive with dengue compared to those from earlier variants (58, 59).

Quantitative differences in binding/neutralization have been documented. In a 2022 cohort, virtually all COVID-19 convalescents had dengue-cross-reactive antibodies (often without prior DENV exposure) (42). However, by 2022–2023 (Omicron era), only ~41.5% of COVID-19 sera showed dengue cross-reactivity, while the remaining ~58.5% were “DENV-non-cross-reactive” (60). Crucially, Omicron-era cross-reactive sera (the 41.5% subset) neutralized all DENV serotypes (1–4), mirroring the broad cross-neutralization seen with earlier variants (60). In contrast, the Omicron-era non-cross-reactive sera still cross-neutralized DENV-1, -2, and -4 to some extent but *failed* to neutralize DENV-3, instead enhancing DENV-3 infection via ADE (evidenced by significantly higher DENV-3 viral titers in cell culture) (60). This serotype-specific loss of neutralization (predominantly affecting DENV-3) is a consistent pattern: Mallick and colleagues observed that earlier variant antibodies (Alpha/Delta) were cross-reactive enough to neutralize even DENV-3, whereas Omicron variant antibodies lacked this capacity, correlating with a global surge in DENV-3 cases during the Omicron wave (58, 59). Emerging data from other groups (including preprints) further support these trends. For example, there are indications that the Omicron variant (or Omicron-specific boosters) elicit lower titers of dengue-cross-reactive antibodies than prior variants or vaccines targeting earlier strains (61). Overall, the pattern that arises is that successive SARS-CoV-2 variants, especially Omicron, have progressively reduced serological cross-reactivity to DENV, with specific gaps such as markedly diminished cross-neutralization of DENV-3 by Omicron-era antibodies (58, 59). Such findings underscore how viral evolution under selective pressure may alter immunological crosstalk between COVID-19 and dengue.

### Implications for surveillance and research gaps

The pandemic reshaped the ecological and epidemiological context for both viruses. COVID-19 interventions (lockdowns, travel restrictions, masking) sharply reduced dengue transmission, acting as a bottleneck or selective sweep. An estimated 0.72 million fewer dengue cases occurred globally in 2020, with many countries seeing 40–60% lower incidence and some experiencing complete seasonal suppression (33, 46). This likely reduced viral genetic diversity and relaxed selection, while creating an “immunity gap” as fewer people acquired dengue immunity, potentially fueling larger outbreaks when transmission resumed. As mobility returned, rare or newly introduced strains could spread rapidly in naive populations, as seen with post-2021 resurgences of specific DENV serotypes. Prolonged lapses in mosquito control may also have favored vector–virus pairs adapted to peri-domestic settings.

For SARS-CoV-2, variant emergence (Alpha, Delta, Omicron, etc.) has been driven mainly by immune pressure from infections and vaccination. Whether dengue-specific factors influenced its evolution is unclear, though in dengue-endemic regions, baseline immune status from tropical infections could affect which variants spread. No evidence indicates that SARS-CoV-2 evolved differently in areas with high dengue incidence, but the question remains open. Epidemiologically, some studies report increased severe dengue after COVID-19 waves (62), suggesting possible effects via immune suppression or healthcare strain. Early speculation that dengue exposure might protect against COVID-19 has been refuted by cohort data showing the opposite: that prior dengue increases COVID-19 risk (50, 57). Unresolved questions include whether co-infection drives viral mutation within hosts (e.g., higher SARS-CoV-2 load or diversity) and whether flavivirus-immune populations shape SARS-CoV-2 antigenic sites over time. The evolutionary and genetic consequences of dengue–COVID-19 co-infection remain poorly defined and require targeted study.

## Influence on clinical severity and immune response evolution

### Clinical severity

Co-infection is linked to more severe outcomes than single infections (16, 50). Systematic analyses report frequent complications, including shock, ARDS, severe organ involvement, and death. A systematic review found high rates of thrombocytopenia and dyspnea, with some cases progressing to septic shock and multi-organ failure (16). In Colombia, concurrent DENV–SARS-CoV-2 infection significantly increased severe dengue manifestations (hemoconcentration, hypotension, mucosal bleeding, persistent vomiting) meeting hemorrhagic fever or “severe dengue” criteria more often than in typical dengue. These patients were also more likely to develop dengue shock syndrome and other warning signs, indicating COVID-19 can worsen dengue’s course (63). Conversely, in Singapore, adults with recent dengue had a 3.3-fold higher risk of severe COVID-19 outcomes when later infected with SARS-CoV-2 (64). This bidirectional aggravation likely reflects compounded inflammation and cumulative organ strain, underscoring the need for aggressive monitoring and supportive care in dengue–COVID co-morbidity.

The clinical severity of co-infection stems from complex immunological crosstalk. Both SARS-CoV-2 and DENV elicit strong innate and adaptive responses, dysregulate Type I interferon pathways, and can trigger cytokine storms and autoantibody production; combined, these effects may amplify hyperinflammation. A key concern is antibody-dependent enhancement (ADE) and cross-reactivity. In dengue, non-neutralizing antibodies from prior infection can facilitate viral entry into Fc-receptor–bearing cells, worsening the disease. This raises the question: could dengue antibodies enhance SARS-CoV-2 infection, or vice versa? Evidence is mixed. Pre-existing anti-DENV IgG has been hypothesized to bind SARS-CoV-2 and exacerbate COVID-19 via ADE (62), supported by epidemiological patterns and mechanistic parallels between flavivirus and coronavirus antibodies. Conversely, Cheng et al. found SARS-CoV-2–elicited antibodies (notably anti–Spike RBD) cross-react with DENV antigens, neutralize DENV *in vitro*, and reduce dengue-induced vascular leak in mice (39). Sera from COVID-19 patients also showed DENV-neutralizing activity. In contrast, a non-peer-reviewed report suggests SARS-CoV-2 antibodies could enhance DENV-2 infection via ADE. Whether dengue antibodies intensify COVID-19 remains under investigation; the Singapore cohort indicates worse COVID-19 outcomes in dengue-immune individuals (64), but direct immunological proof is lacking.

Overall, cross-reactive antibodies and T cells may confer partial cross-protection in some contexts yet worsen disease in others. This duality, alongside the potential for co-infection to trigger both dengue’s vascular leak and SARS-CoV-2’s hyperinflammatory lung injury, underscores the need to clarify how one virus modulates immune responses to the other.

### Eco-epidemiological dynamics and outbreak management

The interplay between dengue and COVID-19 extends beyond biology to epidemiological and public health dynamics. Measures implemented to control COVID-19 have profoundly impacted dengue epidemics, illustrating the intertwined nature of these two diseases at the population level. Strict COVID-19 control policies, such as lockdowns, social distancing measures, school closures, and travel bans, led to marked declines in dengue cases in many regions during 2020. Reduced human movement led to fewer interactions between humans and mosquito vectors in workplaces, schools, and transit areas, thereby lowering dengue transmission. A statistical study in Latin America and Southeast Asia confirmed significantly fewer dengue cases in places with strong mobility restrictions, suggesting human mobility and congregation sites (e.g., schools, markets) are critical drivers of dengue spread, an insight gained incidentally through the COVID experience. These findings indicate that future vector-control approaches should target community gathering sites (47, 65).

COVID-19 measures had dual eco-epidemiological effects. Concurrent epidemics strained healthcare systems, particularly in resource-limited settings, diverting resources from dengue control. Many low- and middle-income countries reassigned staff, hospital beds, and labs to COVID-19, reducing dengue surveillance and vector control (breeding site removal, insecticide spraying, community education). Fear of COVID-19 led some febrile patients to avoid hospitals, delaying dengue diagnosis and treatment. Symptom overlap and lab overload raised concerns about misdiagnosis (66). Cross-reactivity in tests has been documented: SARS-CoV-2 can cause false-positive dengue serology, and dengue antibodies can cause false-positive COVID-19 antibody results (51). Such confusion risks inappropriate management, e.g., IV fluids for presumed dengue in COVID-19 pneumonia or steroids for presumed COVID-19 in dengue, both of which are potentially harmful.

These challenges highlight the need for integrated disease management during syndemics. Some regions acted proactively; in Sri Lanka, outreach campaigns urged febrile patients to seek dengue testing, and despite fewer cases, severe dengue and death rates did not rise in 2020, indicating reduced transmission rather than significant under-reporting (65). Concurrent dengue and COVID-19 outbreaks strained fragile healthcare systems (49), with resource-limited settings facing COVID-19 surges alongside seasonal dengue. Responses included establishing shared fever clinics and maintaining blood bank supplies for severe dengue, despite reduced donor numbers during lockdowns. Clinical care required balancing aggressive fluids for dengue shock with fluid restriction in severe COVID-19. Key lessons include sustaining vector control during respiratory pandemics and strengthening surveillance to detect spikes promptly.

The eco-epidemiological interaction also has socioeconomic dimensions: lockdowns reduced dengue transmission in many regions, yet as restrictions lifted, the potential for dengue resurgence increased. Since 2021, some countries have experienced dengue outbreaks, likely due to accumulated population susceptibility and the reintroduction of the virus through travel (67, 68). Thus, the dengue–COVID syndemic demonstrated that disease control strategies are interconnected; interventions targeting one disease can have both beneficial and adverse collateral effects on another, highlighting the necessity of holistic approaches that address both pathogens simultaneously, particularly in low-resource contexts.

**Figure 1:**
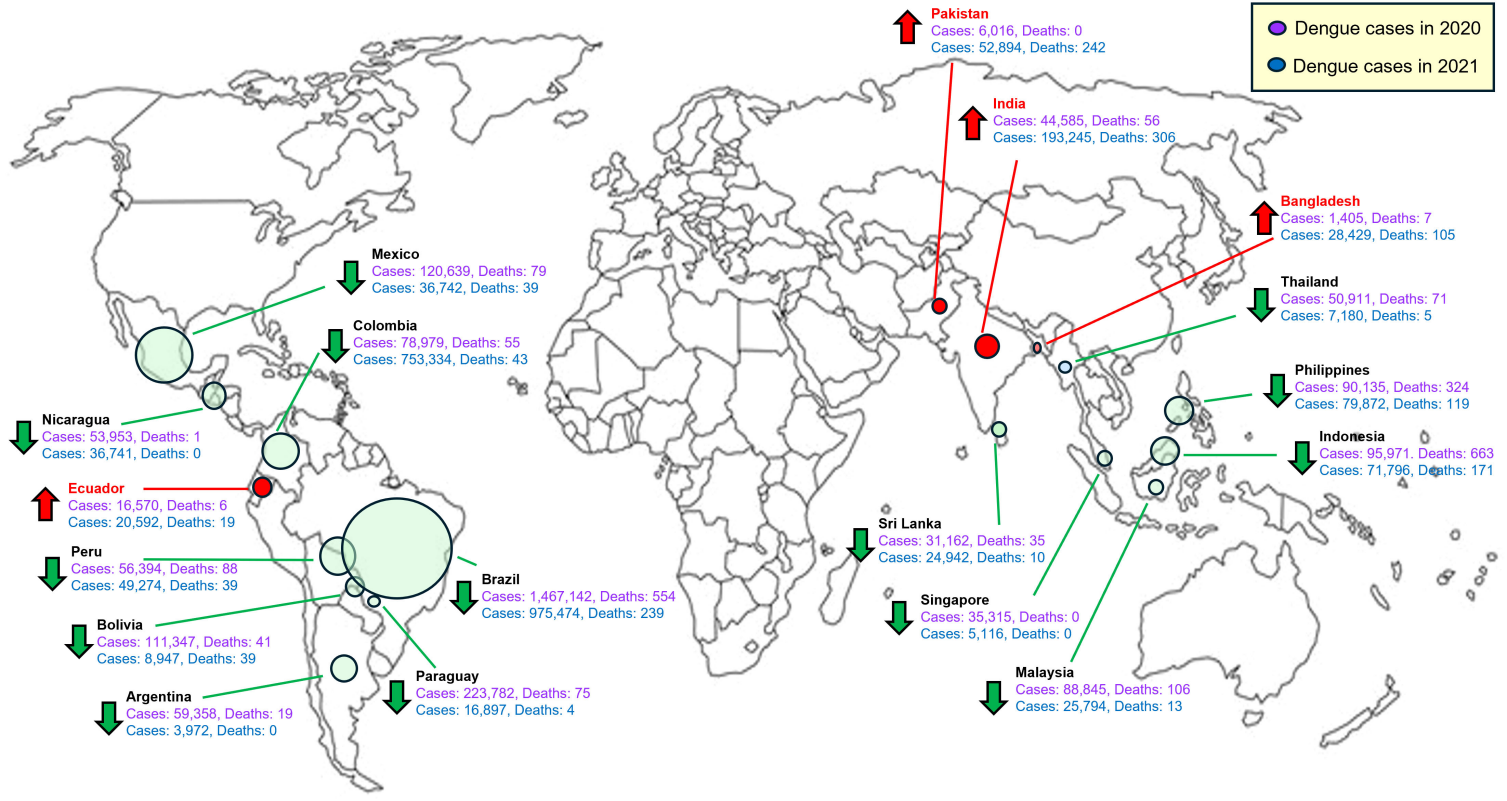
Selected dengue-endemic countries across Latin America and Asia and their dynamics of dengue cases during the COVID-19 pandemic (2020–2021). The map illustrates dengue case trends in selected countries during the COVID-19 pandemic years, highlighting changes between 2020 (purple numbers and circles) and 2021 (blue numbers and circles). Countries marked with red arrows experienced an increase in dengue cases, whereas countries marked with green arrows experienced a decrease. The size of the circle correlates with the number of dengue cases reported. Specific annual dengue case numbers and deaths are detailed for each country, with purple representing 2020 data and blue representing 2021 data. Countries were chosen based on the availability of consistent dengue surveillance data for both years; therefore, some dengue-endemic regions (e.g., parts of Africa) are not represented. This visualization illustrates how COVID-19 pandemic measures, including lockdowns and reduced mobility, affected global dengue transmission dynamics, leading to both declines and resurgences. Modified from (69).

As shown in **Figure 1**, the global distribution map illustrates how COVID-19 pandemic interventions influenced dengue transmission patterns worldwide. Countries marked green experienced significant reductions in dengue cases, attributed to stringent mobility restrictions and lockdown measures that indirectly reduced human-vector interactions. Conversely, nations highlighted in red reported increases, potentially due to disrupted vector control or altered human behaviors, such as increased household water storage during prolonged lockdowns. This visual evidence highlights the intricate interplay between COVID-19 public health interventions and dengue epidemiology, underscoring the importance of integrating epidemiological data and targeted vector-control strategies to address dual epidemics effectively (33, 65, 67, 69).

It is worth noting that **Figure 1** presents aggregated national epidemiological data that illustrate broad trends in dengue incidence during the COVID-19 pandemic (2020–2021). In contrast, **Table 1** summarizes individual clinical cases and smaller cohort studies of dengue–COVID–19 coinfection. These differences reflect distinct purposes and analytical scales rather than inconsistencies: **Table 1** provides detailed clinical insights at the individual-case level, whereas **Figure 1** emphasizes population-level epidemiological trends derived from national surveillance data (3, 13, 14, 16, 69).

### Future research directions and unresolved questions

Our critical appraisal of dengue–COVID–19 co-infection reveals numerous gaps in understanding, which we propose as priorities for future investigation:

#### Mechanistic studies of immune crosstalk

In-depth immunological studies are needed to determine how co-infection alters host responses at the cellular and molecular levels. Key questions include whether dengue predisposes to ADE in COVID-19 or vice versa, the immune cell phenotypes and cytokine profiles unique to co-infection, and whether shared DENV–SARS-CoV-2 T cell epitopes drive cross-reactive responses that mitigate or worsen disease. Addressing these will require controlled cohorts and experimental co-infection models. Given conflicting evidence on cross-neutralization versus enhancement (51, 62), future research should assess the prevalence and clinical impact of cross-reactive antibodies in co-circulating regions and explore vaccine implications, such as whether dengue immunity affects COVID-19 vaccine efficacy or COVID vaccination alters dengue risk.

#### Clinical management and therapeutic strategies

From a clinical research standpoint, more evidence is needed to guide the treatment of co-infected patients. Case reports provide insights, but we lack consensus guidelines: Should thresholds for interventions (like platelet transfusion, steroid use, or ICU admission) be modified for co-infections? Would anti-inflammatory therapies used in COVID-19 (dexamethasone, IL-6 inhibitors) be beneficial or harmful in the context of dengue co-infection? Could antivirals for one virus affect the course of another? Multi-center studies or even clinical trials in endemic regions could help establish the best practices. Additionally, diagnostic research should focus on improving rapid dual testing, for instance, developing combined point-of-care tests that simultaneously screen for SARS-CoV-2 and DENV antigens or genes to facilitate early detection of co-infection. This would mitigate misdiagnosis and ensure timely, appropriate therapy (16, 18, 26, 28, 50).

#### Evolutionary and genomic analysis

To address evolutionary questions, researchers should sequence both viruses from co-infected patients to detect mutations or quasispecies patterns arising under co-infection pressure. Large-scale genomic surveillance in dengue-endemic areas before, during, and after COVID-19 could show whether the pandemic altered DENV lineage replacement or diversity. A key hypothesis is that post-pandemic outbreaks may be driven by variants exploiting the immunity gap or by new lineages introduced after travel resumed (61). Similarly, assessing whether regional SARS-CoV-2 variants correlate with dengue prevalence could offer insights. Such work will clarify how co-circulation shapes pathogen evolution and support predictive models for future syndemics.

#### Eco-epidemiological modeling and health systems research

Future research should use integrated disease models combining vector dynamics, human mobility, and viral transmission to predict outcomes under different interventions (47, 48, 70, 71). Such models can guide contingency planning for controlling both diseases if another respiratory pandemic hits dengue-endemic regions. Investigating health system responses, how hospitals triaged co-infected cases, and how public messaging raised awareness of dual risks will also be valuable. Operational research in resource-limited settings could identify cost-effective strategies to strengthen surveillance, such as integrating community health workers to check for dengue during COVID-19 contact tracing, and to maintain vector control during pandemics.

#### Vaccine and therapeutic interactions

With vaccines now available for COVID-19 (and two licensed dengue vaccines, CYD-TDV (Dengvaxia^®^) and TAK-003 (Qdenga^®^), each with their respective pros and cons: Dengvaxia^®^ is approved only for individuals with prior laboratory-confirmed dengue infection due to an increased risk of severe disease in seronegative recipients, but it offers strong protection against symptomatic and severe dengue in seropositive individuals (72, 73); Qdenga^®^ is indicated for both dengue-naïve and previously infected individuals in certain regions, providing broad serotype coverage, although long-term effectiveness and rare safety signals are still under monitoring along with additional candidates in development), a future research avenue is understanding the interplay between vaccination and pre-existing immunity (74, 75). Does COVID-19 vaccination in dengue-experienced populations impact subsequent dengue infections or diagnostics (for instance, via cross-reactive antibodies)? Conversely, in individuals who receive a dengue vaccine (such as Dengvaxia^®^, Qdenga^®^, or future candidates), is their immune response to a later COVID-19 infection or vaccine any different? Such studies would inform immunization policies in regions affected by both diseases. Likewise, exploring broadly neutralizing antibodies or antiviral drugs that could potentially target both viruses (even indirectly, such as through boosting innate immunity) might be worthwhile, though challenging given that the viruses are from different families (47, 70, 76).

Interdisciplinary research consortia are essential to address the dengue–COVID-19 syndemic, which spans virology, immunology, epidemiology, and public health. Our review shows that co-infection worsens clinical severity, involves complex immune modulation (including potential ADE), and is shaped by ecological forces, while significant knowledge gaps remain. Targeted research will not only clarify this syndemic but also improve preparedness for future overlapping epidemics. Understanding how two distinct viruses interact within hosts and populations offers broader insight into the principles driving modern infectious disease syndemics, an urgent need in an evolving global health landscape.

## Conclusions

Dengue and SARS-CoV-2 co-infection present a major global health challenge, especially in resource-limited tropical regions. Clinical outcomes range from mild illness to shock, multi-organ failure, and high mortality, driven by complex immune interactions, including the poorly understood role of ADE. Evidence for ADE’s clinical relevance remains inconclusive, underscoring the need for targeted mechanistic studies.

Serological cross-reactivity between the viruses creates diagnostic confusion, reinforcing the need for simultaneous molecular testing in co-circulation regions. COVID-19 control measures have also influenced dengue transmission, revealing opportunities for integrated disease management.

This review synthesizes current knowledge on epidemiology, clinical severity, immunopathogenesis, and diagnostic challenges, while outlining key research gaps and priorities. Strengthening interdisciplinary collaboration among clinicians, epidemiologists, immunologists, and policymakers will be essential to manage and mitigate the impacts of concurrent dengue and COVID-19 outbreaks.
